# Degradation Law and Experimental Study of High- Performance Shotcrete Under the Coupling Effect of Sulfate and Chloride Salt

**DOI:** 10.3390/ma18194505

**Published:** 2025-09-27

**Authors:** Jianyu Yang, Senrui Deng, Guanglin Li, Xujun Dai

**Affiliations:** 1 School of Hydraulic and Ocean Engineering, Changsha University of Science & Technology, Changsha 410114, China; 2 School of Civil and Environmental Engineering, Changsha University of Science & Technology, Changsha 410114, China; 3 Department of Commerce, China Construction Fifth Engineering Division Corp, Changsha 410114, China

**Keywords:** high-performance shotcrete, sulfate, chloride salt, durability, erosion

## Abstract

Shotcrete used in underground structures like tunnels is susceptible to sulfate and chloride erosion. In order to systematically study the deterioration law and mechanism of the durability of high-performance shotcrete under a salt erosion environment, the durability test of high-performance shotcrete was carried out by an indoor long-term immersion test using a clear water solution, Na_2_SO_4_ solution, and Na_2_SO_4_ and NaCl mixed solution as erosion mediums. A comparative study was conducted on the effects of different curing time, erosion time, erosion medium, and erosion direction on the physical and mechanical properties and SO_4_^2−^ content. The microstructure was analyzed to reveal the time evolution process and mechanism of the durability of high-performance shotcrete under coupled erosion. The results show the following: (1) The mass change rate of high-performance shotcrete under the action of coupling erosion increases first, then decreases, and then increases. The compressive strength of the surface perpendicular to the jet direction is better than that of the surface along the vertical jet direction. (2) The diffusion depth of SO_4_^2−^ along the injection direction is larger, and the content of SO_4_^2−^ is larger at the same depth. The existence of Cl^−^ delays the diffusion of SO_4_^2−^ to a certain extent. (3) In the early stage of erosion, the corrosion expansion products generated by the external SO_4_^2−^ entering the concrete will fill the original pores and cracks, which improves the durability of the concrete. In the late stages of erosion, the accumulation of corrosion products increases, which accelerates the deterioration of its durability.

## 1. Introduction

High-performance shotcrete is a kind of concrete with excellent mechanical properties, durability, and long-term performance, which is formed by selecting high-quality conventional raw materials; adopting a lower water-binder ratio and optimizing mix ratio; and sending concrete mixtures of cementitious materials, aggregates and water, in a certain proportion to the spraying equipment, and then sending them to the nozzle and accelerator by means of compressed air or other power. After mixing, it is sprayed at high speed onto the sprayed surface, which is referred to as HPSC [1]. Due to the high final setting speed, short time, and high early strength of high-performance shotcrete, it is widely used in underground culverts, modern tunnel lining structures, mine roadway support, damage structure repair, and reinforcement [2,3].

In many environments, there are high concentrations of sulfate ions, chloride ions, and other aggressive ions. The outer surface of high-performance shotcrete in service is directly in contact with the external erosion environment, and the external aggressive ions diffuse into the concrete and undergo complex physical and chemical reactions. Zhang found that with the increase in the sulfate solution concentration and water–cement ratio, the sulfate ion concentration and corrosion depth in concrete increased [4]. These reactions will destroy the original pore structure inside the concrete, resulting in cracking and large-scale spalling of the protective layer, which will eventually degrade the mechanical properties and durability of the service structure [5].

At present, a large number of scholars around the world have carried out lots of research on the durability degradation law and mechanism of ordinary concrete in corrosive salt solution. Chen found through experimental research that mineral admixtures, such as grinding granular blasted slag and fly ash, can improve the ability of concrete to resist sulfate–chloride combined erosion [6]. Luo found that many Na_2_SO_4_ crystals were precipitated on the concrete surface under the coupling erosion of sulfate and chloride salt, and based on the transport mechanism of Cl^−^ in concrete, a machine learning prediction model of Cl^−^ concentration was established. [7]. Li Tao found that the concentration of sulfate ions in concrete increased with the increase in erosion time by carrying out a sulfate corrosion test [8]. Liu Zanqun studied the failure mechanism of the concrete water evaporation zone and proved that carbonization is the cause of physical crystallization of sodium sulfate in concrete [9]. Feng et al. studied the degradation law and service life prediction of tunnel lining concrete with different mineral admixtures under the coupling action of sulfate attack (SA) and dry–wet cycle (DW). The results show that the anti-degradation coefficient of compressive strength of ordinary Portland cement, fly ash, and ground blast furnace slag concrete decreases linearly with the increase in sulfate concentration [10]. Zhang et al. conducted direct shear tests on shotcrete specimens under sulfate attack. The results show that the mechanical behavior of shotcrete specimens under direct shear is significantly affected by sulfate attack. For example, under the same normal stress conditions, the peak shear stress of the shotcrete specimen decreases with the erosion time [11]. Chen et al. studied the deterioration law of concrete durability under the coupling effect of salt corrosion and dry–wet cycle. The damage mechanism of concrete was studied by means of nuclear magnetic resonance, computer tomography, and micro-morphology analysis. The results showed that during the whole erosion process, needle-like erosion products were formed in the pores, and the crystals gradually filled the pores, resulting in expansion pressure on the pore wall, and concrete damage gradually occurred [12]. Li carried out ion erosion experiments and found that the mass and relative dynamic elastic modulus of concrete specimens after erosion in sulfate and sulfate and chloride mixtures increased first and then decreased. The deterioration degree of concrete performance is significantly lower than that after the dry–wet cycle in a single solution after soaking in a mixed solution. And the chloride ion and sulfate ion in mixed solution can inhibit the diffusion of other ions [13]. Xu et al. studied the corrosion of concrete in the presence of sulfate and chloride, and found that the presence of sulfate ions can not only effectively inhibit the diffusion of chloride ions in concrete, but also significantly reduce the concentration of free chloride ions and total chloride ions in concrete [14]. Chen found that in the early stage of erosion, SO_4_^2−^ and Cl^−^ promoted the production of more AFt in concrete, resulting in the filling of original cracks and the reduction in pore volume. As the curing continued, due to the expansion of AFt, the opposite situation gradually appeared in concrete [15]. Hu et al. studied the corrosion degradation mechanism of basalt-fiber-reinforced cast-in-place concrete under the action of sulfate, chloride, and combined attack through experiments. It was found that the filling and bridging effects of basalt fiber reduced the negative effects caused by sulfate–chloride compound erosion [16]. Wang et al. found through comparative experiments that under the erosion of chloride–sulfate–magnesium salt composite solution, the main mineral phases of shotcrete include brucite, gypsum, and ettringite in the early stage of corrosion, and crystal salt is formed in the later stage of corrosion. In ordinary concrete, crystal salt appeared in the early stage of corrosion [17]. Zhao et al. found that strength loss is a typical characteristic of chemical sulfate attack of shotcrete, while the deterioration caused by physical sulfate attack is mainly surface spalling. The presence of chloride ions can alleviate these deteriorations as a whole. At the same time, the shotcrete samples with a low w/b ratio show good resistance to chemical and physical sulfate attack due to their high compactness, low porosity, and permeability [18]. Inaty et al. analyzed the single and combined chemical erosion of sulfate ions and chloride ions on cementitious materials. It was found that chloride ions delayed the formation of ettringite, and the presence of sulfate ions accelerated the attack of chlorides by limiting the formation of Friedel‘s salt [19].

In summary, at present, domestic and foreign scholars have conducted a lot of work on the durability of ordinary concrete in corrosive salt solution and have achieved relatively mature results. However, due to the special composition of HPSC and the incorporation of various admixtures, such as accelerators, its hydration and hardening process and microscopic pore structure have undergone significant changes; therefore, the mechanical properties of ordinary concrete, the hydration and hardening process, the microscopic pore structure, and other related research cannot be fully applied to HPSC. At present, there are few studies on the degradation mechanism and laws of HPSC. Research on the degradation mechanism and law of high-performance shotcrete under the coupling of multiple factors especially needs to be deepened.

In order to make up for this research gap, this paper starts by adapting to the complex service environment of high-performance shotcrete, and uses a 10% Na_2_SO_4_ solution, a 10% Na_2_SO_4_ + 5% NaCl composite solution, and a clear water solution as erosion media, respectively. The durability test of high-performance shotcrete was carried out by long-term indoor immersion, and the durability degradation law of high-performance shotcrete was studied by taking the macroscopic physical and mechanical properties, such as the mass change rate of a specimen and the corrosion resistance coefficient of compressive strength, as indexes.

## 2. Materials and Methods

### 2.1. Materials and Mix of HPSC

High-performance shotcrete raw materials use high-performance shotcrete premix; The cementitious material is P·II 52.5 Portland cement, mixed with a certain amount of silica fume and fly ash; the aggregate is quartz sand with a particle size between 0.15 mm and 2.36 mm. The water reducing agent is a polycarboxylic acid system high-performance water reducing agent (PCE) with a pH value of 7.8, water content of 1.2%, and water reducing rate ≥ 30%. The fiber is polypropylene fiber with a length of 3 mm. The final setting time of concrete is not more than 40 min. The specific match is shown in Table 1.

### 2.2. Preparation of HPSC Specimens

**Figure 1:**
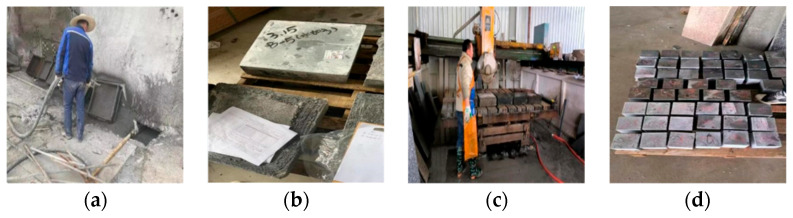
Fabrication of HPSC specimens: (**a**) spraying-forming; (**b**) demolding and curing; (**c**) cutting of specimens; (**d**) specimen completed.

High-performance shotcrete is made by the wet spraying method. The production process is as follows (Figure 1):

Pour the pre-mixed material into the mixer, add water in proportion and stir it fully and slowly in the mixer for 6 min, and transport the mixed wet mixture to the nozzle through the pressure conveying pump and conveying pipe. The injection pressure is adjusted to 0.4 MPa, and the injection volume is controlled at 3–4 m^3^/h.

The steel template with a size of 450 mm×450 mm×120 mm is placed at an inclined angle of 75° with the ground. The nozzle and the lower surface of the test die are kept 90 degrees vertical and kept at a distance of 0.8–1.2 m.

Using artificial spraying, after spraying the large plate, the excess concrete on the surface of the test mold is quickly scraped off, and the surface of the test mold is leveled.

After spraying, sprayed concrete specimens shall not move within 3h, and should be sprinkled and covered.

The large plate was demolded after 8 h of forming and curing. Some of the large plates were cut into cubes, with a side length of 100 mm, by an infrared positioning rock cutting machine immediately after demolding, and the indoor long-term erosion immersion test was carried out. Some of them continued to be cured in the natural environment for 7 days and were then cut into cubes with a side length of 100 mm; then the specimens were standardly cured for 28 days for an indoor long-term immersion test.

### 2.3. Experimental Methods

#### 2.3.1. Long-Term Immersion Test of High-Performance Shotcrete

**Figure 2:**
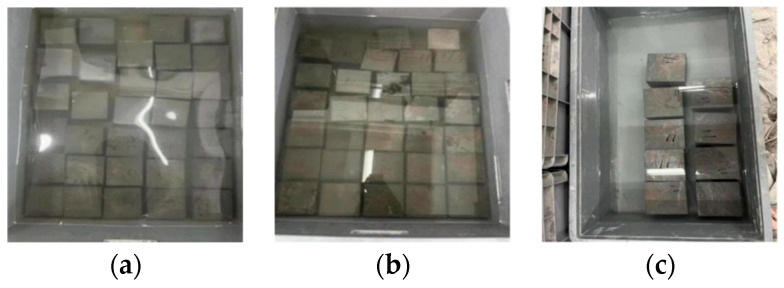
HPSC corrosion solution immersion diagram: (**a**) 10% Na_2_SO_4_ solution; (**b**) 10% Na_2_SO_4_ + 5% NaCl composite solution; (**c**) clear water solution.

The indoor long-term soaking scheme was adopted in the experiment: the soaking solutions were 10% Na_2_SO_4_ solution, 10% Na_2_SO_4_ + 5% NaCl composite solution, and clear water solution (Figure 2). The soaking age was set to 30 d, 120 d, 180 d, 210 d, 240 d, 270 d, 300 d, and 360 d. During the test, the pH value of the soaking solution was measured every 7 days, and the pH value was adjusted to near neutral. The soaking solution was uniformly replaced every 30 days.

After reaching the corresponding test age, the mass loss rate and cube compressive strength of concrete were recorded, and the microstructure of erosion products was characterized. The mass change rate is calculated according to Formula (1). SEM scanning samples of 2–3 mm were taken from the specimen.

(1) $$\omega =\frac{m_{i}-m_{0}}{m_{0}}\times 100\%$$

where *ω* is the rate of mass loss of the eroded concrete cube specimen; *m*_0_ is the initial dry mass of the concrete cube specimen; and *m_i_* is the dry mass of the concrete cube specimen after i days of erosion. The soaking solution is replaced uniformly every day.

#### 2.3.2. SO_4_^2−^ Erosion Experiment

Sulfate content is determined by the standard BaSO4 gravimetric method, and its accuracy and reproducibility are widely recognized [20,21]. In this test, a 100 mm cube test block was used. In order to ensure single-sided erosion, a one-dimensional infiltration mode was adopted (the other five surfaces of concrete were sealed with epoxy resin, and only one surface contact corrosion solution was retained). The test ages were 30 d, 120 d, 180 d, 210 d, 240 d, 270 d, 300 d, and 360 d.

After reaching the corresponding age, along the direction of the erosion surface, 0–2 mm, 2–4 mm, 4–7 mm, 7–11 mm, 11–15 mm (a total of 5 layers) samples of powder (sample particle size is less than 0.1 mm) were scraped, the concrete internal ion content test was conducted in strict accordance with specifications, and sulfate ion content was determined using the barium sulfate gravimetric method, according to Formula (2).

(2) $$W=\left(\frac{M_{X}\times \left(G_{3}-G_{2}\right)}{M_{\mathrm{BaSO}4}}\times \frac{1}{G_{1}}\right)\times 100\%$$

where w is the percentage of the mass of the ions to be tested in the concrete to the mass of the concrete powder, accurate to 0.001%; MX is the molar mass of sulfate ion, 96 g/mol; $G_{1}$ is the mass of concrete powder used in soaking, 0.0001 g; $G_{2}$ is the mass of corundum crucible after burning at 550 °C, 0.0001 g; and $G_{3}$ is the total mass of barium sulfate precipitate and crucible after burning, 0.0001 g.

## 3. Results and Discussion

### 3.1. Durability Degradation Law of HPSC

#### 3.1.1. Quality Loss Rate

**Figure 3:**
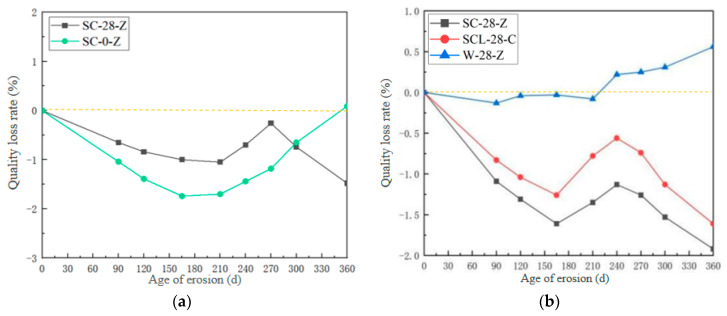
Variation of mass change rate of HPSC with corrosion age: (**a**) different conservation times; (**b**) different erosion solutions.

The variation of mass loss rate of high-performance shotcrete with age under different erosion solutions and different curing times was obtained (Figure 3). Compared with different curing times, the sulfate erosion uncured test group (SC-0-Z) showed a trend of decreasing first and then increasing, and the sulfate erosion curing 28-day test group (SC-28-Z) showed a trend of decreasing first and then increasing and then decreasing. Compared with different erosion solutions, the sulfate–chloride coupling erosion test group (SCL-28-Z) showed a trend of decreasing first, then increasing and then decreasing; the overall trend showed a gradual downward trend. The water control group (W-28-Z) showed a slow upward trend. The reason is that the concrete is directly in contact with the erosion medium without curing. At this time, the internal hydration reaction of the concrete has not been completed, the internal pore structure is still growing, and the external erosion medium is more likely to spread inside, forming more expansive salt products. The corrosion resistance is worse, and the cracking and spalling are obvious in the later stage, so the mass loss rate increases in the later stage of erosion. There are chloride ions and sulfate ions in the sulfate–chloride salt composite salt solution. Chloride ions first enter the concrete to form chloride ion erosion products, which refine the pore structure and hinder the entry of sulfate ions. Chloride ion erosion products and sulfate ion corrosion products are converted to each other, so that the two reach an equilibrium state, and the mass loss rate gradually decreases. Different from the quality decline of ordinary concrete due to cracking and spalling in the later stage of corrosion, the high-performance shotcrete after standard curing does not show a lot of slag and skin spalling in the later stage of the test due to its excellent raw materials and doped polypropylene fibers. Therefore, the quality increases with the accumulation of erosion products in the cracks.

#### 3.1.2. Compressive Strength

**Figure 4:**
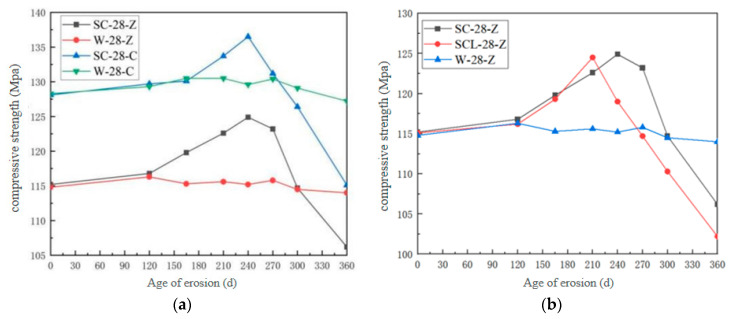
Variation of compressive strength of HPSC with corrosion age: (**a**) different erosion directions; (**b**) different erosion solutions.

Figure 4 shows the variation of compressive strength of high-performance shotcrete with erosion age under different erosion solutions and different erosion surfaces. And with the increase in erosion time, the compressive strength of high-performance shotcrete increases first and then decreases. When the intrusion surface is different, the maximum compressive strength of sulfate immersion along the jet direction test group (SC-28-Z) and the sulfate immersion vertical jet direction test group (SC-28-C) in 240 days is 124.9 MPa and 136.5 MPa, respectively. Under different erosion solutions, the maximum compressive strength of the sulfate–chlorine salt composite solution immersion test group (SCL-28-Z) and the clear water solution immersion test group (W-28-Z) were 121.5 MPa and 116.0 MPa, respectively. Under different directions, the compressive strength of SC-28-C is greater than that of SC-28-Z, which is due to the special molding process of shotcrete. In the production of shotcrete, the high-performance shotcrete mixture is sprayed onto the large plate at high speed by wind pressure. The wind pressure squeezes the mixture to both sides and accumulates continuously, while the concrete mixture along the sprayed wind pressure surface will rebound after impact and gradually accumulate and form. A large number of bubbles and cracks parallel to the jet direction will be carried along the jet direction, resulting in cracks and pores with the direction of the jet surface, so the compressive strength of the vertical jet direction surface is higher, and the resistance to sulfate attack is stronger.

Under different erosion solutions, sulfate ions and chloride ions coexist in the composite salt solution. Since chloride ions first diffuse into the shotcrete and react with cement hydration products, they first form Friedel’s salt and increase the compactness of the concrete. The formation of Friedel’s salt slows down the early erosion rate of concrete, and the Friedel’s salt formed by the reaction of the chloride ion and the Aft formed by the reaction of the sulfate ion will interact with each other. The diffusion coefficient of the chloride ion is about twice that of the sulfate ion. Chloride ions first bind to aluminum to form Friedel’s salt, which will reduce the chance of sulfate ions binding to aluminum to form Aft. Moreover, the solubility of Aft in the chloride solution is three times higher than that in clear water, which also reduces the formation of Aft, so the final amount of erosion products is less than that of sulfate erosion. And the figure shows that the increase rate of compressive strength of SCL-28-Z is less than that of SC-28-Z, which indicates that chloride salt will improve the sulfate resistance of high-performance shotcrete.

### 3.2. Study on the Erosion Law of SO_4_^2−^ in HPSC

**Figure 5:**
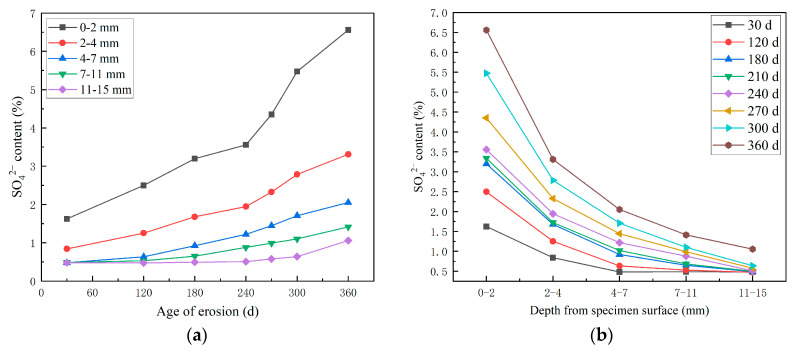
Sulfate ion content distribution curve of sulfate attack HPSC: (**a**) SO_4_^2−^ change pattern with time; (**b**) SO_4_^2−^ change pattern with depth.

Figure 5 is the test results of SO_4_^2−^ concentration of high-performance shotcrete under long-term immersion in sulfate solution, which shows that the SO_4_^2−^ concentration at each test depth gradually increased with the increase in erosion time. The SO_4_^2−^ concentration increased rapidly in the early stage of erosion. At 180 days to 240 days, the diffusion rate slowed down. After 240 days of erosion, the SO_4_^2−^ concentration increased again.

This is due to the fact that in the early stage of erosion, there are original pores and cracks in the high-performance shotcrete, and the external SO_4_^2−^ diffuses into the concrete through the concentration difference, resulting in the increase in SO_4_^2−^ content. With the deepening of the erosion time, the external SO_4_^2−^ enters and reacts with the cement hydration products and unhydrated cement particles inside the concrete. The generated corrosion products gradually fill the original cracks and pores inside the shotcrete, making the concrete denser, hindering the entry of SO_4_^2−^, resulting in a slower increase rate of SO_4_^2−^ concentration. In the later stage of erosion, the SO_4_^2−^ entered by the outside world gradually increases, and the generated corrosive products also gradually increase. The formation of new pores and cracks provides a channel for the outside SO_4_^2−^ to enter the concrete, so the increase rate of SO_4_^2-^ concentration becomes larger. The analysis of SO_4_^2−^ concentration at different depths shows that the closer to the surface of the specimen, the higher the SO_4_^2−^ concentration, and the SO_4_^2−^ content from the surface layer (0–2 mm) is the largest, and the SO_4_^2−^ content in the second layer (2–4 mm) decreases rapidly. The SO_4_^2−^ content also decreases with the increase in depth from the surface layer of the specimen; it is a gradual decrease and tends to be gentle. This is because the external SO_4_^2−^ diffuses into the concrete through the concentration difference. The closer to the surface of the specimen, the greater the SO_4_^2−^ concentration difference with the outside world, and the greater the SO_4_^2−^ content. Secondly, because the sulfate invades the high-performance shotcrete, it reacts with the hydration product to form an expansive product to fill the pores, which hinders the inward diffusion of the sulfate. Therefore, the deeper the distance from the surface of the specimen, the lower the SO_4_^2−^ content.

#### 3.2.1. Study on the SO_4_^2−^ Erosion Law of HPSC Along the Direction of Shotcrete and Perpendicularly to the Direction of Shotcrete

**Figure 6:**
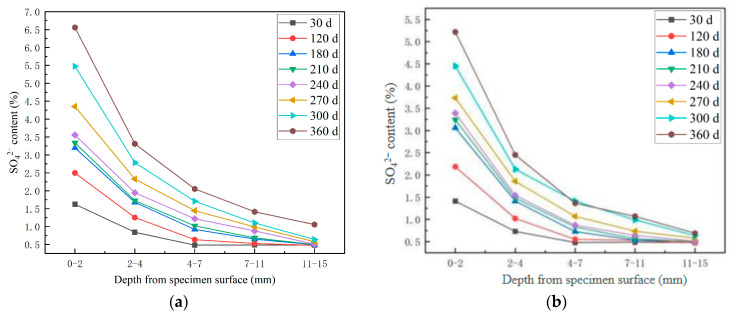
Distribution curve of sulfate content of HPSC with erosion depth under different erosion directions: (**a**) frontal erosion of SO_4_^2−^ along the spray surface; (**b**) lateral erosion of SO_4_^2−^ along the spray surface.

Figure 6 shows the test results of SO_4_^2−^ content of high-performance shotcrete under the condition of long-term immersion of sulfate solution along the normal spray surface and the side spray surface. It can be seen from Figure 6 that the SO_4_^2−^ diffusion distribution law of high-performance shotcrete along the spray direction surface and vertical spray direction surface has similarities and differences. The similarities are as follows: (1) The maximum erosion depth increases with the increase in erosion age. (2) The content of SO_4_^2−^ in each depth increases with the increase in erosion age. (3) The deeper the depth from the surface of the specimen, the lower the SO_4_^2−^ content, and the decrease in SO_4_^2−^ content between 0–2 mm and 2–4 mm from the surface of the specimen is the largest. The differences are as follows: (1) The content of SO_4_^2−^ in each depth of side erosion is lower than that of front erosion. (2) The SO_4_^2−^ decline rate of 0–2 mm and 2–4 mm away from the surface of the specimen under side erosion is greater than that under front erosion. (3) The diffusion rate of side erosion is lower than that of front erosion. The reason is analyzed as follows: High-performance shotcrete sprays the concrete slurry jet duct through high-strength wind pressure. After the large plate is sprayed, only the excess concrete is scraped off, and there is no vibration process. The wind pressure during spraying has the effect of squeezing and rebounding the large plate concrete. The generated cracks and pores will be more parallel to the positive spray surface. As the aggressive product gradually accumulates, it fills and squeezes the pore wall, causing pore expansion and crack penetration, generating more cracks parallel to the positive spray surface, which is more conducive to the diffusion of external ions into the concrete.

#### 3.2.2. Study on the Erosion Pattern of SO_4_^2−^ in a Complex Salt Solution Environment

**Figure 7:**
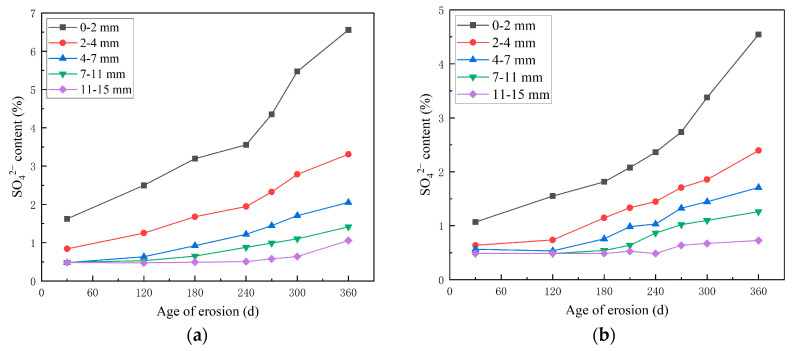
Distribution curve of sulfate content of HPSC with age under different corrosion solutions: (**a**) SO_4_^2−^ solution; (**b**) SO_4_^2−^ and Cl^−^ composite solution.

Figure 7 shows the SO_4_^2−^ content test results of high-performance shotcrete under the erosion of sulfate solution and sulfate–chloride compound solution. From Figure 7, the SO_4_^2−^ content under the erosion of composite salt solution is lower than that under the erosion of sulfate solution during the whole erosion age. When immersed for 120 days, the SO_4_^2−^ content at a depth of 0–2 mm from the surface of the specimen under the erosion of composite solution is much lower than that of sulfate solution erosion by 37.9%. This is because when the concrete is eroded by chloride and sulfate at the same time, the Cl^−^ and SO_4_^2−^ diffused from the outside world will react with the aluminum phase (Al(OH)^4−^) in the concrete pore solution to form Friedel’s salt and ettringite, respectively. However, the total content of the aluminum phase in concrete is limited. Therefore, there is a competitive relationship between Cl^−^ and SO_4_^2−^ in the process of chemical bonding. Because the ionic radius of Cl^−^ is smaller than that of SO_4_^2−^, Cl^−^ is easier to enter the concrete than SO_4_^2−^, and forms Friedel’s salt, which fills the original pores and cracks and hinders the entry of SO_4_^2−^ in large quantities. SO_4_^2−^ in turn reacts with Cl^−^ to inhibit the formation of Friedel’s salt, thereby reducing the amount of chloride binding. Therefore, in the early stage, Cl^−^ will significantly reduce the diffusion rate of SO_4_^2−^. With the deepening of erosion time, a large amount of Friedel’s salt and ettringite will accumulate inside the concrete, and the expansion damage caused by sulfate is related to the content of calcium aluminate in the concrete, but Cl^−^ will react with the expansive substance of monosulfide calcium sulphoaluminate, which consumes the expansive product generated by SO_4_^2−^ to a certain extent, so the diffusion rate of SO_4_^2−^ increases in the later stage.

### 3.3. SEM Scanning Electron Microscope Analysis and EDS Energy Spectrum Analysis

**Figure 8:**
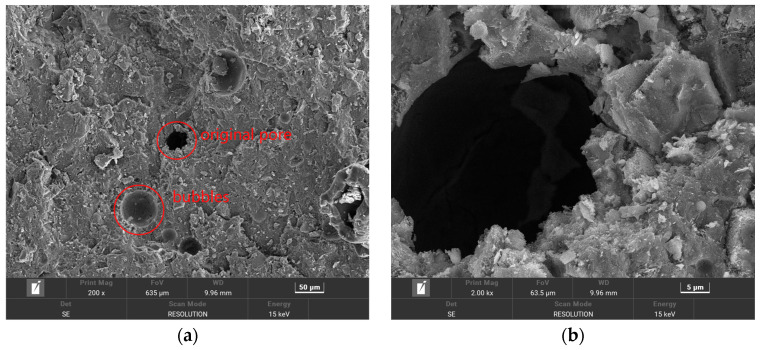

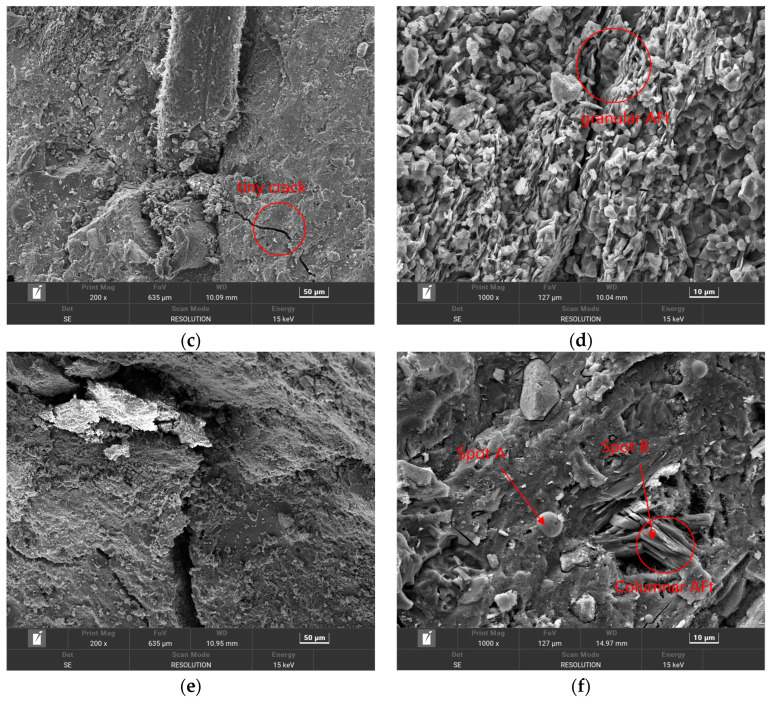
Microstructure of surface layer of HPSC under different erosion ages under sulfate solution erosion: (**a**) without erosion; (**b**) without erosion; (**c**) after 180 days of erosion; (**d**) after 180 days of erosion; (**e**) after 360 days of erosion; (**f**) after 360 days of erosion.

**Figure 9:**
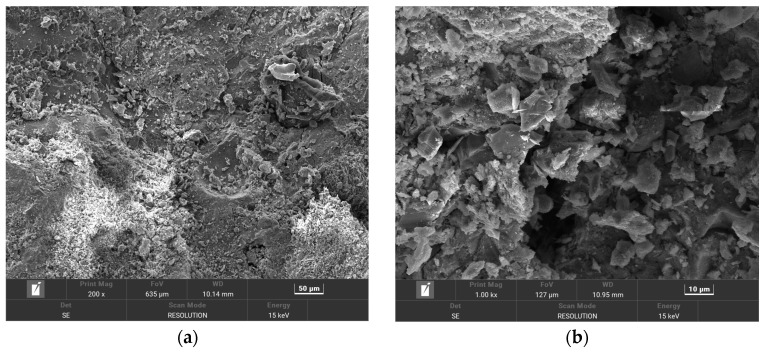

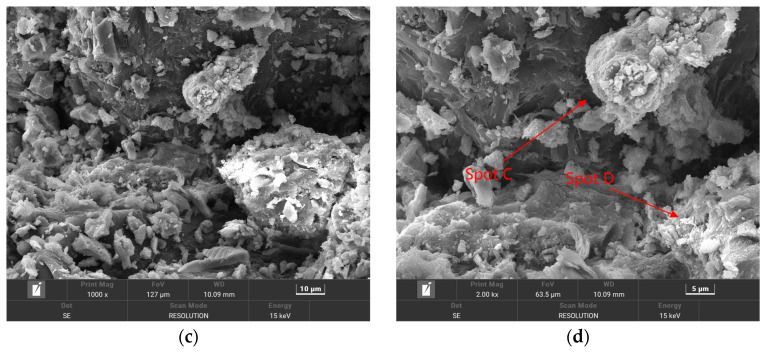
Microstructure of surface layer of HPSC under different erosion ages under sulfate–chloride solution erosion: (**a**) after 180 days of erosion; (**b**) after 180 days of erosion; (**c**) after 360 days of erosion; (**d**) after 360 days of erosion.

**Figure 10:**
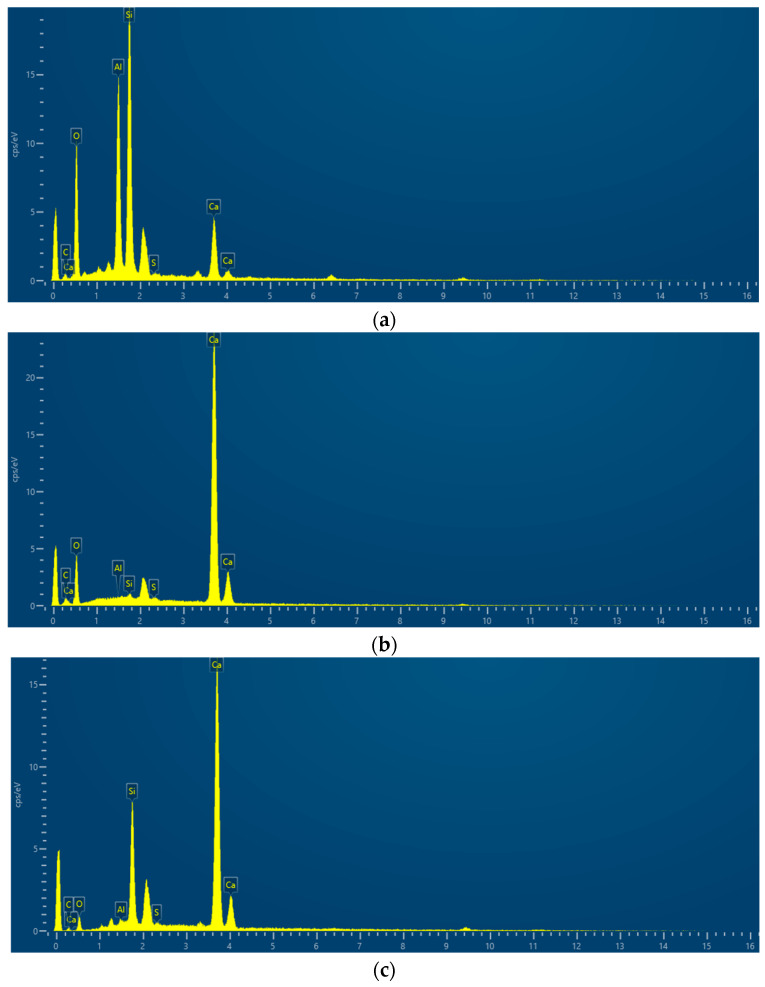

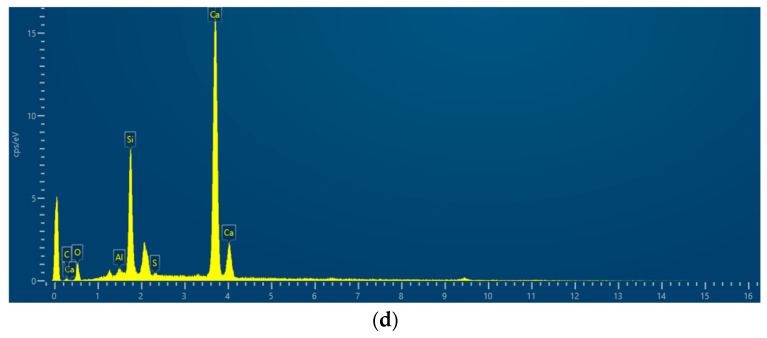
EDS energy spectrum analysis diagram: (**a**) Spot A; (**b**) Spot B; (**c**) Spot C; (**d**) Spot D.

In order to make the microstructure of HPSC in different erosion solutions have obvious contrast, HPSC immersed in sodium sulfate solution alone and sulfate–chlorine salt composite solution were selected for electron microscope scanning tests. The microstructure of HPSC with erosion cycles of 0 d, 180 d, and 360 d is given below, using scanning electron microscopy (Figure 8 and Figure 9) and EDS energy spectrum analysis (Figure 10).

#### 3.3.1. Analysis of Microstructure of HPSC Under Sulfate Solution Erosion

From Figure 8a,b, it can be seen that the surface of the HPSC specimen immersed is very smooth and clean, and the integrity and compactness of the hydrated calcium silicate gel (C-S-H) are good. Therefore, a large number of erosion products are not seen in the microstructure of the HPSC without immersion. It can be seen from Figure 8c,d that the corrosion products of HPSC samples increased significantly after 180 days of sulfate solution erosion. At this time, the original defects inside the concrete were basically filled, but no new cracks appeared. This may be due to the extremely high strength of HPSC itself, and the expansion internal stress generated by the accumulation of corrosion products did not break through the strength of the concrete. On the contrary, the accumulation of erosion products not only improves the compactness of the concrete but also hinders the continuous entry of external erosion ions into the concrete. Therefore, before 210 days of erosion, the compressive strength of HPSC increases with the increase in erosion time, and the ion diffusion rate gradually decreases. It can be seen from Figure 8e,f that when the erosion age reaches 360 d, there are obvious cracks inside HPSC, the surface of the concrete becomes loose and ulcerated, the density and integrity of hydrated calcium silicate gel (C-S-H) decrease, and a large number of needle-shaped ettringite are accompanied around some pores and cracks. The formation of a large amount of ettringite is the root cause of cracks. The new cracks generated further do not provide channels for the external erosion ions, and then generate more erosion products, and promote the formation of new cracks. The cycle is repeated, and the cracks inside the concrete are gradually connected to each other, and the concrete loses its original strength.

#### 3.3.2. Micromorphology Analysis of HPSC Under Sulfate–Chloride Composite Solution Erosion

In Figure 9c,d, we can see that compared with before erosion, it can be clearly seen that the surface flatness of HPSC decreased after 180 days of erosion, and the concrete matrix began to loosen and fester, and a small amount of erosion products appeared on the surface. Compared with the local amplification diagram (Figure 8d and Figure 9d), the erosion products generated after 180 days of sulfate–chloride mixed solution erosion were significantly less than those after 180 days of sulfate erosion. This is because in the composite salt solution environment, Cl^−^ will always react with the cement hydration products inside the concrete, and the corrosion products generated by Cl^−^ itself will compact the inside of the concrete to prevent the entry of SO_4_^2−^. This also verifies the relevant degradation rules of HPSC macroscopic mechanical properties and ion diffusion. It can be seen from Figure 9b,c that a large amount of Friedel’s salt and cauliflower-like ettringite were generated inside HPSC after 360 days of erosion in sulfate–chloride mixed solution. These erosion products will accumulate in most of the cracks and pore structures inside the specimen and then show the expansion of original cracks and the increase in new micro-cracks. The compactness of concrete decreases, and a large number of salt crystals appear at the cracks. Comparing the micro-morphology of sulfate attack at the same age, it can be seen that ettringite exhibits a large cluster-like and rod-like interwoven structure under sulfate attack (Figure 8e,f). After sulfate–chloride coupling erosion, ettringite is mainly in block and cauliflower shapes (Figure 9b,c), and ettringite and Friedel’s salt are interwoven together. This is because under sulfate attack, a large amount of ettringite is formed by the reaction between a large amount of SO_4_^2−^, and the hydration of cement in concrete gradually accumulates. Cl^−^ in the composite solution will affect the reaction between SO_4_^2−^ and concrete.

#### 3.3.3. EDS Analysis

It can be seen from Figure 10 that in Figure 8f, A and B are ettringite and hydrated calcium silicate, respectively. According to the growth shape of ettringite, it can be seen that they are intertwined, interspersed, and filled with the whole pores and cracks. Under the long-term immersion of sulfate solution, due to the cracking of concrete, SO_4_^2−^ is more likely to erode into the interior of concrete in the later stage of erosion, and it continues to develop in pores and cracks to generate more ettringite crystals. With the increase in ettringite crystals, the volume expansion gradually develops into a large piece of coarse rod shape, which in turn promotes the development of cracks. It can be seen from c and d in Figure 10 that the products generated by HPSC after long-term immersion in sulfate-chloride salt are ettringite and Friedel’s salt. With the increase in erosion time, the generated corrosion products will gradually fill the interior until new cracks are generated, or old cracks are expanded, which further provides channels for corrosive ions until the concrete test block is completely degraded.

## 4. Conclusions

In this paper, a 10% Na_2_SO_4_ solution and a 10% Na_2_SO_4_ + 5% NaCl composite solution were selected, and the durability test of high-performance shotcrete eroded by composite salt was carried out by the indoor long-term immersion method. The physical and mechanical properties and the ion diffusion law were studied. The following conclusions are drawn:

(1) The mass change rate of high-performance shotcrete increases first, then decreases, and then increases with the increase in erosion time. Under different jetting surfaces, the compressive strength of the vertical jetting direction surface is better than that along the jetting direction surface, but the superiority of the vertical jetting direction surface gradually weakens in the later stage of erosion, and the chloride salt will improve the resistance to sulfate attack.
(2) With the progress of the erosion process, the content of SO_4_^2−^ and Cl^−^ in concrete increases. Compared with different erosion directions, the SO_4_^2−^ diffusion depth of the ion invasion surface along the jet direction is larger, and the SO_4_^2−^ content is larger at the same depth. Compared with different erosion solutions, the presence of Cl^−^ delays the diffusion of SO_4_^2−^ to a certain extent, and the final diffusion depth of SO_4_^2−^ is smaller under the erosion of composite salt.
(3) The deterioration process of the durability of high-performance shotcrete can be divided into the formation stage of ettringite and gypsum, the filling stage of corrosion products, and the generation stage of new cracks. In the early stage of erosion, the external SO_4_^2−^ diffuses into the concrete to generate corrosion expansion products to fill the original pores and cracks and enhance the durability of high-performance shotcrete. In the middle and late stages of erosion, the accumulation of corrosion products increases, resulting in the expansion of original cracks and the generation of new cracks, which accelerates the deterioration of the durability of high-performance shotcrete.
(4) For high-performance shotcrete eroded by sulfate solution and sulfate–chloride solution, the concentration of erosion solution also has a great influence on the erosion operation of concrete. This study mainly considers a 10% Na_2_SO_4_ solution and a 10% Na_2_SO_4_ + 5% NaCl composite solution as the erosion solutions and does not set different erosion medium concentration gradient combinations. Subsequent research can set different gradient erosion solution concentrations to study the durability degradation law in more detail. In addition, the service environment of HPSC is often more complex, such as sulfate, chloride, magnesium salt, carbonization, and other multi-factor coupling erosion. In the follow-up study, the influence of various factors on the durability degradation of concrete can be considered. In the follow-up study, laboratory immersion and field exposure, the dry–wet cycle test, and other tests can be designed to systematically study the durability of HPSC in a complex environment.

## Figures and Tables

**Table 1:** Mix proportion of HPSC.

HPSC Ingredients	Mix Proportion
Cement	1.0
Water	0.2948
Silica fume	0.1282
Coal ash	0.1025
Fine aggregate	1.282
Water reducing agent	0.0051
Polypropylene fiber	0.1%
Thickener	0.0769

Note: The representation method of mix proportion is relative dosage representation method and Fibers are volume ratios.

## Data Availability

The original contributions presented in this study are included in the article. Further inquiries can be directed to the corresponding author.
